# Inhibition of JNK Aggravates the Recovery of Rat Hearts after Global Ischemia: The Role of Mitochondrial JNK

**DOI:** 10.1371/journal.pone.0113526

**Published:** 2014-11-25

**Authors:** Sehwan Jang, Sabzali Javadov

**Affiliations:** Department of Physiology, School of Medicine, University of Puerto Rico, San Juan, Puerto Rico, United States of America; Faculty of Medicine & Health Sciences, United Arab Emirates

## Abstract

c-Jun N-terminal kinase (JNK), a stress-activated MAPK, is activated during cardiac ischemia-reperfusion (IR). The role of JNK inhibitors in cardioprotection against IR still remains controversial, in part, due to spill-over effects of non-specific inhibitors. In the present study, we sought to examine whether inhibition of JNK by SU3327, a specific JNK inhibitor that inhibits upstream JNK signaling rather than the kinase activity of JNK, improves cardiac function and reduces heart damage during IR. Hearts of male Sprague-Dawley rats perfused by Langendorff were subjected to 25 min of global ischemia followed by 30 min reperfusion in the presence or absence of SU3327. Cardiac function was monitored throughout the perfusion period. Myocardial damage was extrapolated from LDH activity in the coronary effluent. At the end of reperfusion, mitochondria were isolated and used to measure respiration rates and mitochondrial permeability transition pore opening. Protein analysis of mitochondria predictably revealed that SU3327 inhibited JNK phosphorylation. Although SU3327 significantly reduced cell damage during the first minutes of reperfusion, it did not improve cardiac function and, furthermore, reduced the mitochondrial respiratory control index. Interestingly, SU3327 activated the other stress-related MAPK, p38, and greatly increased its translocation to mitochondria. Mitochondrial P-JNK and P-p38 were co-immunoprecipitated with complex III of the electron transfer chain. Thus, JNK plays an essential role in cardiac signaling under both physiological and pathological conditions. Its inhibition by SU3327 during IR aggravates cardiac function. The detrimental effects of JNK inhibition are associated with reciprocal p38 activation and mitochondrial dysfunction.

## Introduction

Heart diseases due to myocardial ischemia, including myocardial infarction and heart failure, are the major causes of death in developed countries, and their prevalence continues to grow [1]. Even if the ischemic period is short or limited, the functional recovery of a reperfused heart is often less successful than expected due to “reperfusion injury” [2]. Indeed, the reperfusion of acutely ischemic myocardium can independently induce cardiomyocyte death [3]–[5]. The major contributing factors of cardiomyocyte death during ischemia-reperfusion (IR) are oxidative stress, calcium overload, mitochondrial permeability transition pore (MPTP) opening, and hypercontracture [5].

JNK, a member of the mitogen-activated protein kinase (MAPK) family, has been implicated in reactive oxygen species (ROS)- and other stress-induced apoptosis [6], [7]. JNK has been shown to be activated *in vivo* and *ex-vivo* models of IR [8] as well as in patients during cardiopulmonary bypass [9] and heart failure [10]. Activation of the JNK pathway is considered an important step in the progression of cell death in response to simulated ischemia [11]. Pharmacological inhibition of JNK decreased cardiomyocyte apoptosis and infarct size from IR [12], [13]. On the other hand, increased JNK activation was shown in preconditioned hearts during IR [14], and protein kinase C-ε (PKCε), which is known to play a crucial role in cardioprotection, was found to interact with mitochondrial JNK [15]. Inhibition of JNK conferred no protection to the anisomycin-induced infarct size [16]. Interestingly, both genetic inhibition and activation of JNK protected the myocardium from IR [17]. These conflicting data underline the complex role of JNK in the heart, in which both its inhibition and activation can confer cardioprotection by different mechanisms, depending on the timing, severity of stress, and type of stimuli.

Translocation of JNK to mitochondria was observed in response to DNA damage [18] and H_2_O_2_- [19] and IR- [20] induced oxidative stress. Interestingly, mitochondrial JNK signaling has been shown to further stimulate ROS generation [20] thus promoting a mitochondrial, JNK-mediated ROS self-amplification loop [21]. Furthermore, Sab, a mitochondrial scaffold of JNK, was found to participate in the translocation of JNK to mitochondria and mitochondrial ROS generation [22].

In this study, we investigated whether inhibition of JNK offers cardioprotection against IR using a Langendorff-mode perfusion of the isolated rat heart. We employed SU3327, which, in contrast to other JNK inhibitors, such as SP600125, inhibits upstream JNK activation rather than the kinase activity of JNK. We found that SU3327 aggravated the recovery of isolated hearts from IR. Moreover, the inhibitor elicited different effects depending on the presence or absence of stress and the timing of administration. Our findings imply the existence of crosstalk between the JNK and p38 pathways in response to oxidative stress, in which downregulation of JNK stimulates p38, which, in turn, aggravates cardiac function. Furthermore, inhibition of JNK during IR enhances interaction of p38 with complex III of the electron transport chain (ETC), which itself can cause cardiac dysfunction.

## Materials and Methods

### Animals

Male Sprague-Dawley rats weighing 225–275 g were purchased from Charles River (Wilmington, MA, USA). All experiments were performed according to protocols approved by the University Animal Care and Use Committee of the UPR Medical Sciences Campus (Approval number: A7620113) and conformed to the *Guide for the Care and Use of Laboratory Animals published by the US National Institutes of Health* (NIH Publication No. 85-23, revised 1996).

### Langendorff-mode heart perfusion and experimental groups

**Figure 1:**
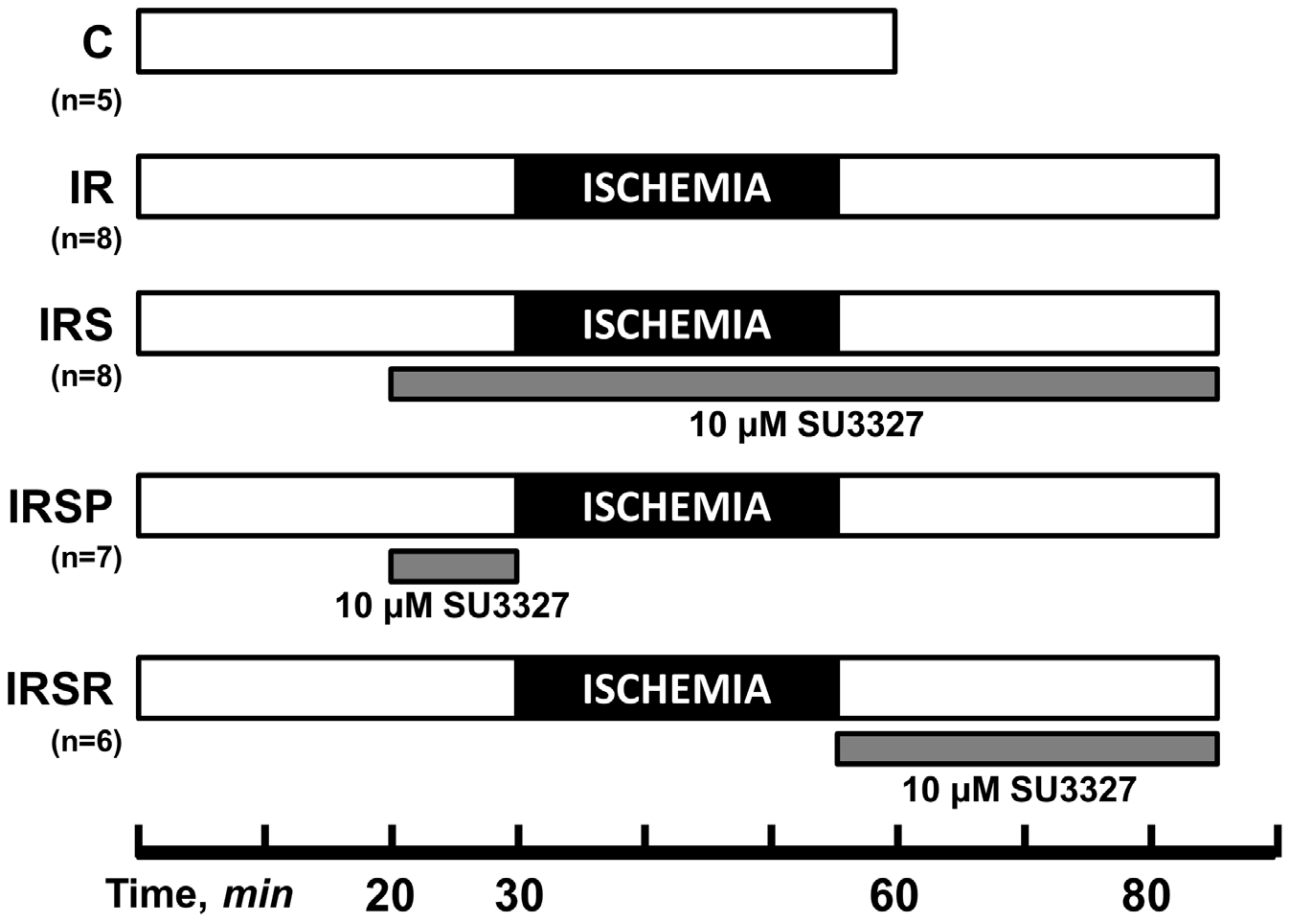
Perfusion protocols. Experimental groups: 1) C, hearts perfused for 60 min (n = 5); 2) IR, hearts subjected to 25-min global ischemia followed by 30-min reperfusion (n = 8); 3) IRS, hearts subjected to global ischemia followed by reperfusion in the presence of 10 µM SU3327 throughout IR (n = 8); 4) IRSP, hearts perfused in the presence of 10 µM SU3327 for 10 min before ischemia, then subjected to global ischemia followed by reperfusion without JNK inhibitor (n = 7), and 5) IRSR, hearts subjected to global ischemia followed by reperfusion with 10 µM SU3327 at reperfusion only (n = 6).

On the day of the experiment, the rats were euthanized with a guillotine in accordance to the *AVMA Guidelines for the Euthanasia of Animals: 2013 Edition*. The rationale for the use of decapitation of conscious rats was to avoid side effects of anesthesia on cardiovascular system, particularly cardiac function, which was an important end-target of the present study. The hearts were rapidly removed, immersed in Krebs solution, and retrogradely perfused via a non-recirculating Langendorff perfusion system at constant flow [23]. A water-filled latex balloon was inserted into the left ventricle for continuous monitoring of heart rate (HR), left ventricular systolic (LVSP) and end diastolic (LVEDP) pressure. Left ventricular developed pressure (LVDP) was calculated as the difference between LVSP and LVEDP (LVDP = LVSP-LVEDP). Cardiac work was estimated by the rate-pressure product (RPP) calculated as RPP = LVDP×heart rate (HR). Measurements were recorded using Labscribe2 (iWorx 308T, Dover, NH, USA) or Chart5 (Powerlab, Colorado Springs, CO, USA). Protocols of perfusion are illustrated schematically in Fig. 1. Animals were randomly assigned to the following treatment groups: (1) C, non-ischemic hearts perfused for 60 min (n = 5); (2) IR, hearts subjected to 25-min global ischemia followed by 30-min reperfusion (n = 8); (3) IRS, hearts subjected to global ischemia followed by reperfusion in the presence of 10 µM SU3327 throughout IR (n = 8); (4) IRSP, hearts perfused in the presence of 10 µM SU3327 for 10 min before ischemia, then subjected to global ischemia and followed by reperfusion without JNK inhibitor (n = 7); (5) IRSR, hearts subjected to global ischemia followed by reperfusion with 10 µM SU3327 at reperfusion only (n = 6). SU3327 was prepared as 50 mM stock in DMSO. Global normothermic ischemia was induced by switching off the pump after a total pre-ischemic period of 25 min, with the heart immersed in buffer maintained at 37°C. In all experiments, an ischemic period of 25 min was followed by 30 min of reperfusion with flow at pre-ischemic levels. Samples of the coronary effluent were collected prior to ischemia and during reperfusion at indicated time points to measure lactate dehydrogenase (LDH) activity. LDH activity was assessed by an enzymatic method as previously described [23] with minor modifications. After the corresponding protocols, the hearts were used to isolate mitochondria.

### Isolation of mitochondria

To isolate mitochondria, the ventricles were cut, weighed and homogenized with a Polytron homogenizer at 1500 rpm for 5 sec in the ice-cold sucrose buffer containing 300 mM sucrose, 10 mM Tris-HCl, and 2 mM EGTA, at pH 7.4. Mitochondria were isolated from the homogenate by centrifugation at 2000×g for 2 min in a benchtop centrifuge to remove cell debris, followed by centrifugation of the supernatant at 10,000×g for 5 min to sediment the mitochondrial suspension. The pellet was then washed two times at 10,000×g for 5 min in 40 mL of BSA-free sucrose buffer. The final pellet was resuspended in 300 µL of sucrose buffer. The yield of mitochondria was 12.1±0.31 mg/mL. A 200-µL sample of the suspension was then used for measurement of mitochondrial respiration rates and MPTP opening. A 100-µL sample was mixed with protease inhibitor cocktail (P8340, Sigma, USA.) and phosphatase inhibitor cocktail (P0044 and P5726, Sigma, USA), then immediately frozen in liquid nitrogen and stored at −80°C for western blot analysis.

### Respiration rate measurements in isolated cardiac mitochondria

Measurement of mitochondrial respiration was performed at 37°C using a YSI Oxygraph (Yellow Springs, OH, USA) model 5300 equipped with a Clark-type oxygen electrode [24]. Measurements were recorded and analyzed using Chart5 (Powerlab, Colorado Springs, CO, USA). Mitochondria were suspended in a buffer containing (in mM) 125 KCl, 20 MOPS, 10 Tris, 0.5 EGTA, and 2 KH_2_PO_4_, at pH 7.2, supplemented with either of the following substrates to measure complex I- and complex II-mediated respiration rates, respectively: (i) 2.5 mM 2-oxoglutarate and 1 mM L-malate or (ii) 2.5 mM succinate and 1 µM rotenone. Respiration rates were measured in the absence (state 2) and presence (state 3) of 1 mM ADP. At the end of each run, 0.5 µM antimycin A followed by 10 mM ascorbate and 0.3 mM N,N,N′,N′-tetramethyl-p-phenylendiamine (TMPD) were added, and the new rate of respiration was measured. The respiratory control index (RCI) was calculated as ratios of respiration in the presence of ADP (state 3) to the absence of ADP (state 2).

### Measurement of MPTP opening in isolated mitochondria

Swelling of de-energized mitochondria as an indicator of MPTP opening in the presence or absence of Ca^2+^ was determined by monitoring the decrease in light scattering at 545 nm as described previously [25]. Freshly isolated mitochondria were incubated at 37°C in 1 mL buffer containing 0.2 M sucrose; 10 mM Tris-MOPS, pH 7.4; 5 mM succinate; 1 mM Pi; 2 µM rotenone; 10 µM EGTA-Tris. Mitochondria containing ∼0.5 mg of protein were added and absorbance was monitored for 2 min, then CaCl_2_ was added to 100 µM and read for another 2 min. Rates of swelling were calculated as the ratio of decrement of absorbance with CaCl_2_ to decrement of absorbance without CaCl_2_.

### Western blot analysis

Immunoblotting was performed as described previously [24]. The antibodies used in this study were against total and phosphorylated forms of p38, JNK, and ERK1/2, as well as COXIV (Cell Signaling Technology, Danvers, MA, USA). The signals were visualized by VersaDoc 4000 Gel Imaging System (Bio-Rad, Hercules, CA, USA) and analyzed by ImageJ [26].

### Protein carbonylation assay

Protein carbonyls were analyzed according to the method described previously [27], [28]. Briefly, an aliquot of the mitochondrial protein was derivatized with dinitrophenylhydrazine (DNPH, Sigma, USA) under acid denaturing conditions. Proteins were separated by SDS-PAGE and subject to immunoblotting with anti-dinitrophenyl primary antibodies (Sigma, USA) at 1∶1000 dilutions. Each sample was loaded with an identical amount of protein (12.5 µg). In order to correct for non-specific binding of the antibodies, aliquots of the mitochondrial proteins that had been acid-denatured but not treated with DNPH were run in parallel. Blots were scanned and carbonylations were determined as intensities for each sample after subtraction of non-specific background signals.

### Co-immunonoprecipitation

Co-immunoprecipitations of P-JNK and P-p38 with the specific antibody to each protein were conducted by following the recommended protocol of Dynabeads (Invitrogen-Life Technologies, Grand Island, NY, USA). A separate aliquot of mitochondrial proteins (0.3 mg each) was incubated with 2 µg of antibody-conjugated beads overnight. The immunoprecipitated proteins were dissolved in Laemmli buffer for immunoblot analysis using the specific antibody against adenine nucleotide translocase (ANT), voltage-dependent anion channel (VDAC), cyclophilin D (CyP-D), and mitochondrial ETC complexes.

### Cell culture

H9c2 embryonic rat cardiomyocytes were purchased from the American Type Culture Collection (Manassas, VA, USA) and cultured according to the manufacturer's recommendations. In short, the cells were cultured in modified DMEM/Ham's F-12 (Invitrogen, Carlsbad, CA, USA) media supplemented with 10% fetal bovine serum, and maintained in 95% air and 5% CO_2_ at 37°C. Prior to all experiments, cells were serum-starved for 24 hrs. Cells with 85∼90% confluence from passages 3∼20 were used for experiments.

### Cell viability

Cell viability was determined using the trypan blue exclusion method. The cells were cultured at a density of 5×10^5^ cells on 100-mm dishes and exposed to 75 µM H_2_O_2_ for 30 min, with or without inhibitors. After treatment, cells were rinsed with PBS, detached with Hyclone Trypsin (Thermo Fisher Scientific, Waltham, MA, USA) and counted using the TC20 Automated Cell Counter (Bio-Rad, Hercules, CA, USA). The per cent of viable and dead cells was calculated from a total number of counted cells.

### Total ROS in cultured cardiomyocytes

Cells were trypsinized with a 0.25% trypsin–EDTA solution (Thermo Scientific HyClone, Logan, UT, USA) and centrifuged at 500×g for 5 min. at room temperature. The resulting pellets were resuspended in culture medium, and cells were incubated with 20 µM 2′7 dichlorofluorescein diacetate (DCF-DA; Alexis Biochemicals) for 30 min at 37°C. The cells were again centrifuged at 500×g for 5 min. to remove medium with excess dye, and the pellets were resuspended in PBS and added to a 96-well plate. Fluorescence intensity was measured using a Spectramax M3 plate reader (Molecular Devices, Sunnyvale, CA, USA) at an excitation of 485 nm and emission of 530 nm.

### Mitochondrial membrane potential in cultured cardiomyocytes

Cells plated in a 24-well culture plate (4×10^5^ cells/well) were incubated for 30 min. with the membrane potential-sensitive dye 5,5′,6,6′-tetraethyl-benzimidazolylcarbocyanine iodide (JC-1, 10 µg/mL, Molecular Probes, Eugene, OR, USA). Afterwards, the intensity of fluorescence was immediately measured using a Spectramax M3 microplate reader (Molecular Devices) at 527 and 590 nm for emission, and 488 nm for excitation.

### Statistical analysis

Values are presented as mean ± SE. Data were analyzed using two-way ANOVA, followed by a post hoc Student's t-test. *P*<0.05 was considered statistically significant.

## Results

### The JNK inhibitor SU3327 affects heart function and cell death

**Figure 2:**
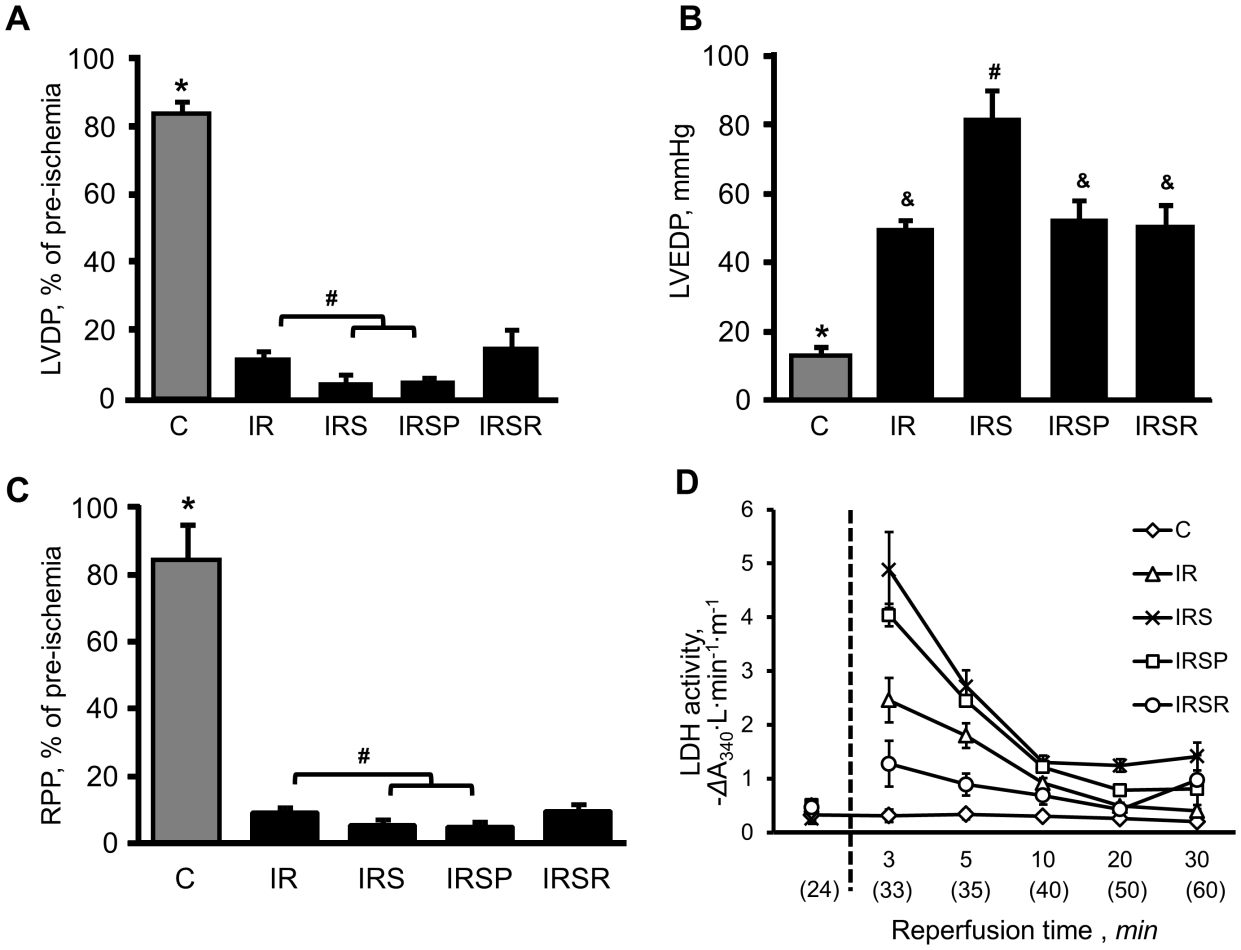
Heart function. (***A***) Left ventricular (LV) developed pressure (LVDP) calculated as the difference between LV systolic pressure (LVSP) and LV end-diastolic pressure (LVEDP). Data are expressed as a percentage of pre-ischemic values. *P<0.0001 C *vs* other groups, ^#^ *P*<0.05 IR *vs* IRS and IRSP. (***B***) LVEDP at the end of reperfusion (for IR, IRS, IRSP and IRSR) or perfusion (C). (***C***) The rate-pressure product (RPP) calculated as RPP = LVDP×HR. RPP recovery during the reperfusion period is shown as percent of pre-ischemia values. (***D***) Lactate dehydrogenase (LDH) activity in the coronary effluent. The dotted line represents 25 min of ischemia for IR, IRS, IRSP, and IRSR groups. Time in parentheses represents perfusion time for the C group (without IR). LDH activity is shown as decrement of absorbance at 340 nm per min for liter of perfusate per gram heart. *P<0.001 C *vs* other groups, #, &: significantly different from the other indicators (*P*<0.05). n = 5 for C, n = 8 for IR, n = 8 for IRS, n = 7 for IRSP, and n = 6 for IRSR groups.

We examined the effects of the JNK inhibitor SU3327 both on hearts subjected to IR and hearts without IR. As shown in (Fig. 2), the addition of SU3327 aggravated the recovery of heart function. However, in the IRSR group, the inhibitor briefly reduced LDH activity and cell death shortly after reperfusion. In the IR group, treatment with SU3327 resulted in a reduction of LVDP values by 90% (*P*<0.05, C *vs* IR) at reperfusion when compared to pre-ischemia (Fig. 2A). Addition of the inhibitor before ischemia aggravated post-ischemic recovery by 50% (*P*<0.05, IR *vs* IRS and IRSP). In the IRS group, LVEDP was elevated by 30 mmHg (*P*<0.05, IR *vs* IRS) (Fig. 2B,C).

Hearts subjected to IR showed increased LDH activity in the coronary perfusate, indicating increased cell death. Interestingly, administration of SU3327 during reperfusion only decreased LDH activity by 40% (*P*<0.05, IR *vs* IRSR; Fig. 2D). However, when SU3327 was applied before ischemia, LDH activity was even greater than IR alone (IR *vs* IRSP).

### Mitochondrial abnormalities induced by SU3327

**Figure 3:**
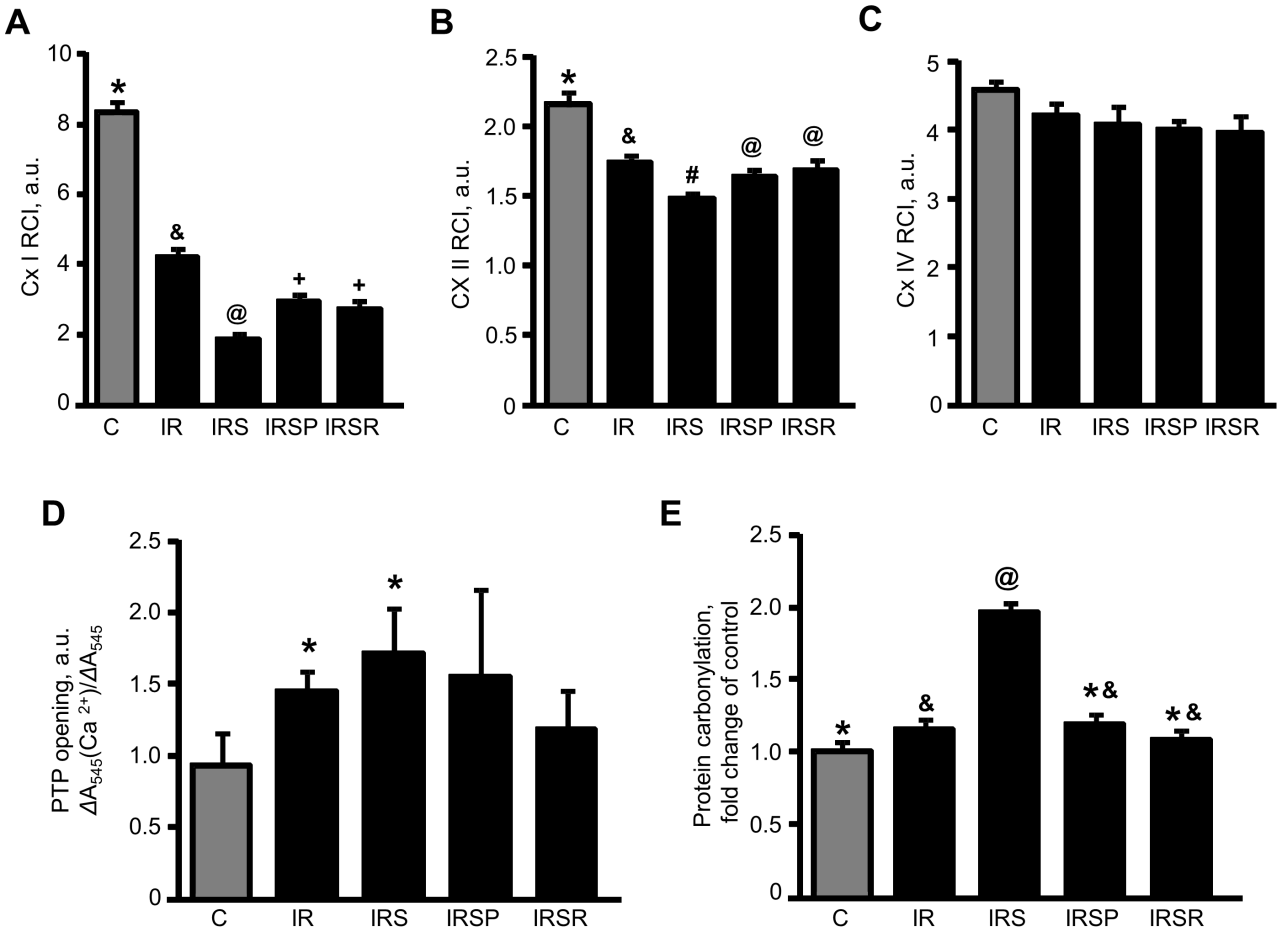
Mitochondrial respiratory control index, MPTP opening and carbonylation of mitochondrial proteins. (***A***) Mitochondrial respiratory control index (RCI) at complex I measured in the presence of 2.5 mM 2-oxoglutarate and 1 mM L-malate substrates. (***B***) RCI at complex II measured in the presence of a 2.5 mM succinate substrate. (***C***) RCI at complex IV measured in the presence of 10 mM ascorbate and 0.3 mM TMPD. *, #, &, @, +: significantly different from the other indicators (*P*<0.05). (***D***) Increment of the rate of mitochondrial swelling by addition of 100 mM CaCl_2_. *: significantly different from group C (*P*<0.05), (***E***) Carbonylation levels of mitochondrial proteins. The data were represented as the ratio of intensities from DNPH-treated samples to non-treated samples compared to control for each group. *, #, &, @: significantly different from the other indicators (*P*<0.05). n = 5 for C, n = 8 for IR, n = 8 for IRS, n = 7 for IRSP, and n = 6 for IRSR groups.

Mitochondria isolated from hearts in all groups were used to determine RCI and MPTP opening. As shown in (Fig. 3), the RCIs of mitochondria at complexes I and II were lower in experimental groups than control groups. IR markedly reduced RCI by 50%, indicating a decrease in the efficiency of respiratory coupling and ATP synthesis. With administration of SU3327, RCIs at complexes I and II were reduced even further than IR alone (*P*<0.05; Fig. 3A,B). However, RCIs at complex IV did not show significant reduction (*P*>0.05; Fig. 3C). Mitochondrial swelling induced by Ca^2+^, an indicator of MPTP opening, was increased by SU3327 (*P*<0.05; Fig. 3D).

Elevated levels of carbonylated proteins are a marker of increased oxidative stress. The levels of oxidized proteins in the mitochondria of the IR group were increased by 1.2 fold compared to control (*P*<0.05, Fig. 3E). The IRSR group showed a 1.7 fold higher (*P*<0.05; IR *vs* IRS) carbonylation of mitochondrial proteins compared to the IR group. There was no difference between IR, IRSP and IRSR groups.

### JNK inhibition affects IR-induced JNK phosphorylation

**Figure 4:**
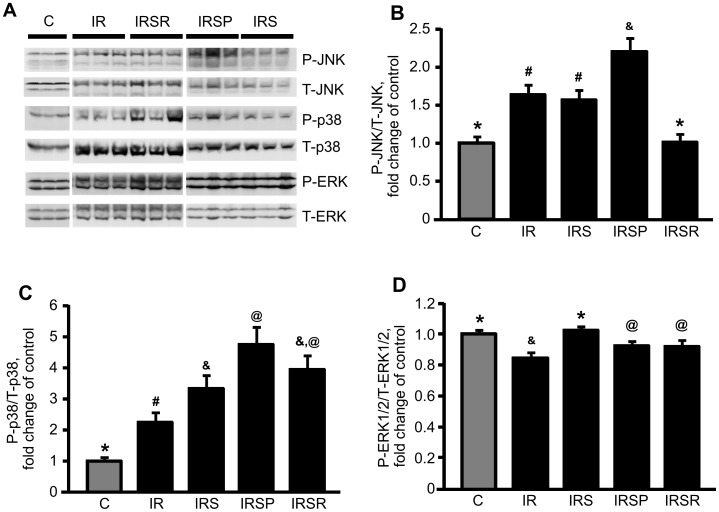
MAPK activation in heart homogenates. Representative Western blots (***A***) and quantitative data of phosphorylated JNK (***B***), p38 (***C***), and ERK1/2 (***D***) were examined by Western blot analysis using phospho-specific antibodies. The data were calculated as the ratio of phosphorylated or total protein levels, and normalized to control for each MAPK. *, #, &, @, +: significantly different from the other indicators (*P*<0.05). n = 5 for C, n = 8 for IR, n = 8 for IRS, n = 7 for IRSP, and n = 6 for IRSR groups.

To determine the effect of SU3327 on MAPKs, we examined the phosphorylation of JNK, as well as p38 and ERK1/2 in IR-hearts treated with SU3327. IR induced activation of JNK and p38, and slightly inhibited ERK. As shown in (Fig. 4), IR activated JNK by 1.5 fold (*P*<0.05, C *vs* IR) and p38 by 2.5 fold (*P*<0.05, C *vs* IR). SU3327 added during reperfusion only induced a 37% (*P*<0.05, IR *vs* IRSR) reduction of JNK phosphorylation. Administration of the inhibitor before ischemia and during reperfusion did not affect activation of JNK (IR *vs* IRS). Furthermore, SU3327 was less able to inhibit JNK when it was added during pre-ischemia only (IR *vs* IRSP).

### JNK inhibition activates p38 and ERK1/2

IR increased p38 activation by 2.5 fold (*P*<0.05, C *vs* IR). All three JNK inhibitor-treated groups demonstrated more activated p38 than the IR group (IR *vs* IRS, IRSP, IRSR) (Fig. 4C). On the other hand, IR reduced ERK1/2 activation. Inhibition of JNK during reperfusion restored ERK1/2 activation (IR *vs* IRS), while ERK1/2 activation was only partially restored in other SU3327 treatment groups (IR *vs* IRSP and IRSR).

### SU3327 affects phosphorylation of mitochondrial p38 and ERK1/2

**Figure 5:**
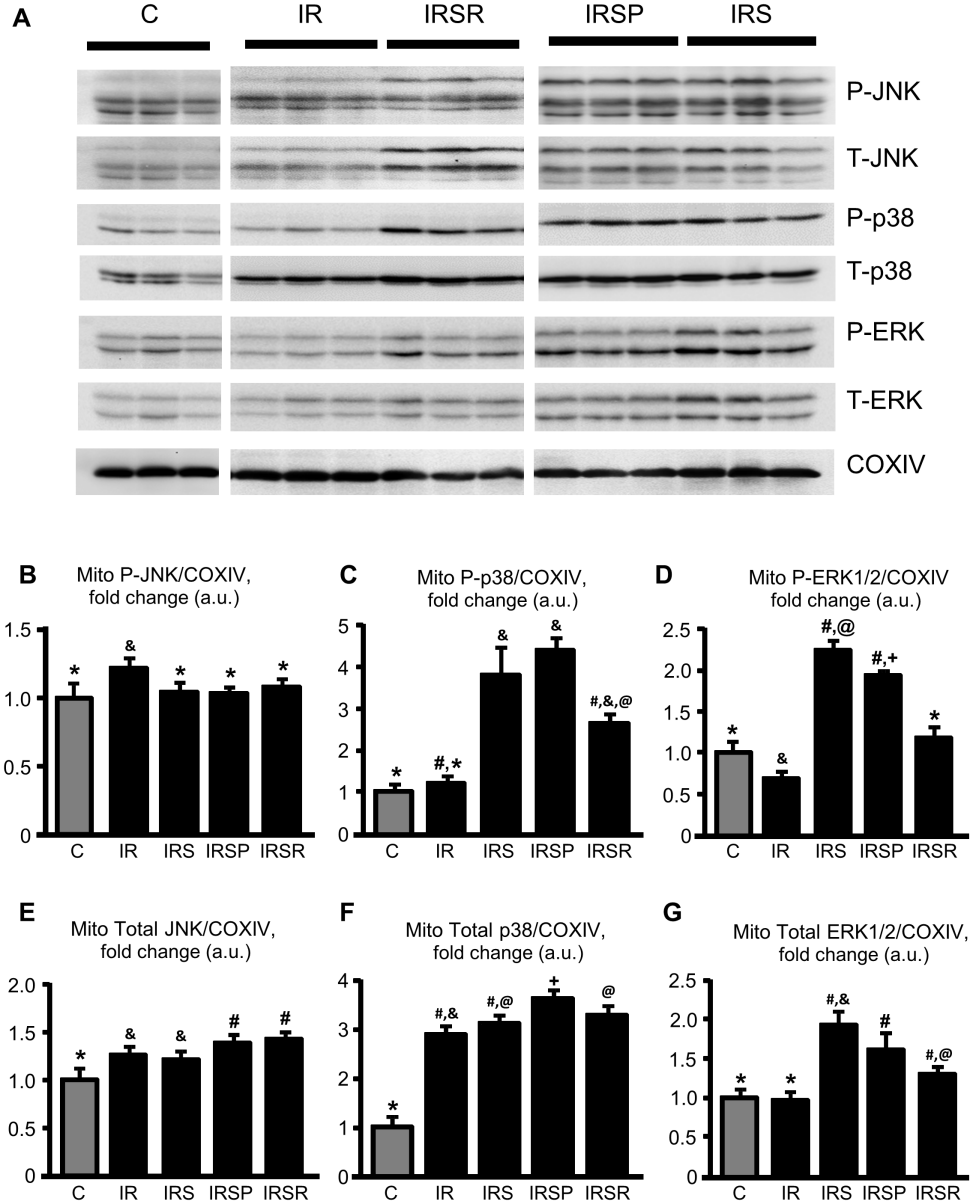
MAPK activation in mitochondrial fractions of isolated hearts. Representative Western blots (***A***) and quantitative data of phosphorylated JNK (***B***), p38 (***C***), and ERK1/2 (***D***), as well as total (***E***) JNK, (***F***) p38, and (***G***) ERK1/2 were calculated as the ratio of phosphorylated or total protein to COXIV, and normalized to control for each MAPK. *, #, &, @, +: significantly different from the other indicators (*P*<0.05). n = 5 for C, n = 8 for IR, n = 8 for IRS, n = 7 for IRSP, and n = 6 for IRSR groups.

To determine the effect of JNK inhibition on the activation of mitochondrial MAPKs, we examined the phosphorylation of JNK, p38 and ERK1/2 in response to IR, in the presence or absence of SU3327 (Fig. 5). Predictably, inhibition of JNK was observed in all SU3327-treated groups. IR activated JNK by 1.2 fold (*P*<0.05) and reduced ERK1/2 activity by 0.7 fold (*P*<0.05), but did not affect p38 activity. Although IR reduced phosphorylation of mitochondrial P-ERK1/2, SU3327 increased P-ERK1/2 levels. Treatment with the inhibitor before ischemia and during reperfusion did not reduce total mitochondrial JNK compared to IR (IR *vs* IRS). We also found that IR increased mitochondrial total JNK and p38 (Fig. 5E,F). Total ERK1/2 was not affected by IR, but all groups treated with SU3327 showed increased total ERK1/2 (Fig. 5G).

### Protein-protein interactions between JNK and mitochondrial proteins

**Figure 6:**
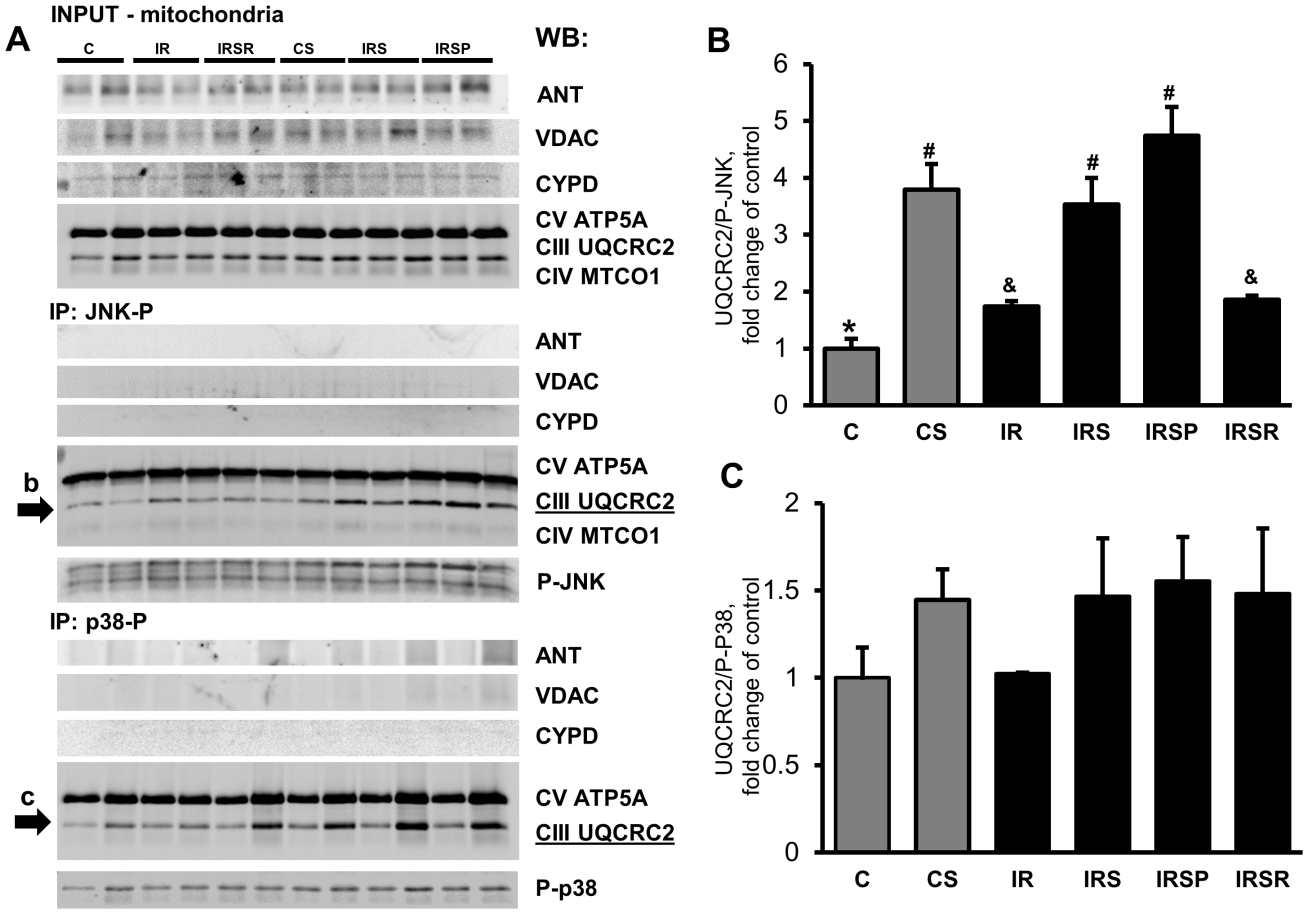
Co-immunoprecipitation of mitochondrial proteins with P-JNK and P-p38. Representative immunoblots showing interaction between P-JNK, P-p38, the MPTP components ANT, VDAC, CyP-D, and components of mitochondrial oxidative phosphorylation. (***A***) Cardiac mitochondria from each group were immunoprecipitated (IP) with P-JNK and P-p38. The complexes were subjected to SDS-PAGE followed by immunoblotting (IB) with indicated antibodies. Bands that underwent densitometry analysis are indicated by arrows (a,b). (***B***) Densitometric data for UQCRC2 (a component of mitochondrial complex III), were normalized to P-JNK. CS, non-ischemic hearts perfused for 60 min with 10 µM SU3327 added at 20 min after beginning of perfusion (n = 4). *, #, &: significantly different from the other indicators (*P*<0.05). (***C***) Densitometric data for UQCRC2 (a component of mitochondrial complex III), normalized to P-p38. n = 3 per group.

To examine the interaction between mitochondrial JNK or p38 and the MPTP complex, we immunoprecipitated mitochondrial proteins with antibodies against P-JNK and P-p38, and then immunoblotted with the MPTP regulators, ANT, VDAC, and CyP-D, as well as mitochondrial ETC complex proteins. As shown in (Fig. 6A), IR increased interactions between activated JNK and UQCRC2, a component of mitochondrial complex III, by 1.7 fold (*P*<0.05 *vs* control). Treatment with SU3327 further increased the interaction, except in the IRSR group, which showed no significant difference. The IRS and IRSP groups showed a 2.0- and a 2.7-fold increase (*P*<0.05 for both) in JNK-UQCRC2 interactions, respectively, compared to the IR group. UQCRC2 was also co-immunoprecipitated with p38, although the differences between groups were not statistically significant (Fig. 6B). Likewise, JNK and p38 interacted with ATP5A, a component of complex V that was not affected by IR in the presence or absence of SU3327. Furthermore, the regulatory proteins of the MPTP complex, ANT, VDAC, and CyP-D, were found in cardiac mitochondria, but they were not detected after co-immunoprecipitation with either P-JNK or P-p38 (Fig. 6A).

### p38 inhibition alleviates the aggravation resulted from JNK inhibition

**Figure 7:**
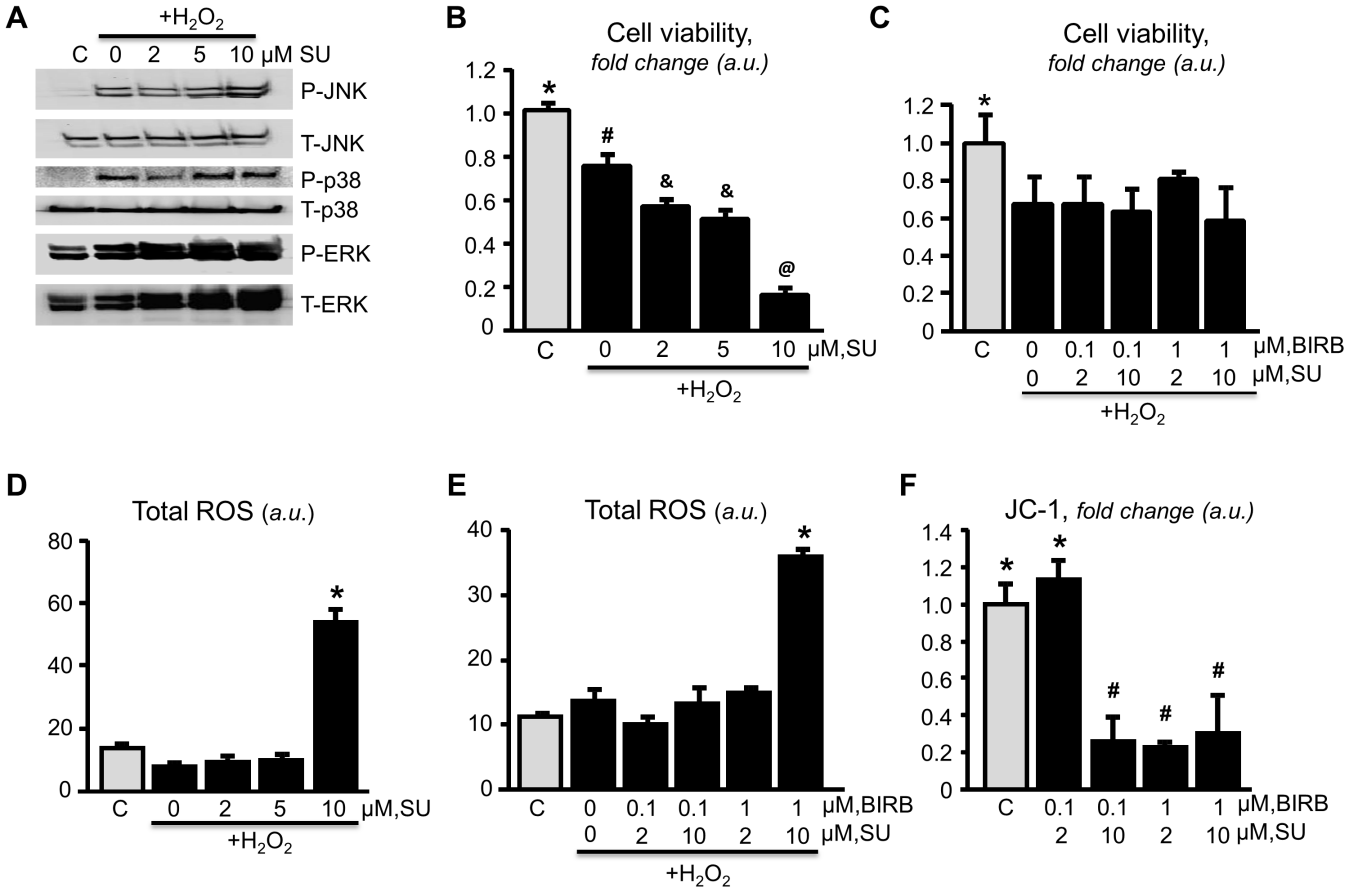
MAPK activation in H9C2 cells. Representative immunoblots showing activation of JNK, p38, and ERK (***A***). Cardiomyocytes were pretreated with JNK inhibitor SU3327 (SU), then treated with 75 µM H_2_O_2_ (***B,D***). Cardiomyocytes were pretreated with SU and p38 inhibitor BIRB796 (BIRB), then treated with 75 µM H_2_O_2_ (***C,E,F***). *, #, &: significantly different from the other indicators (*P*<0.05). n = 6 per group.

**Figure 8:**
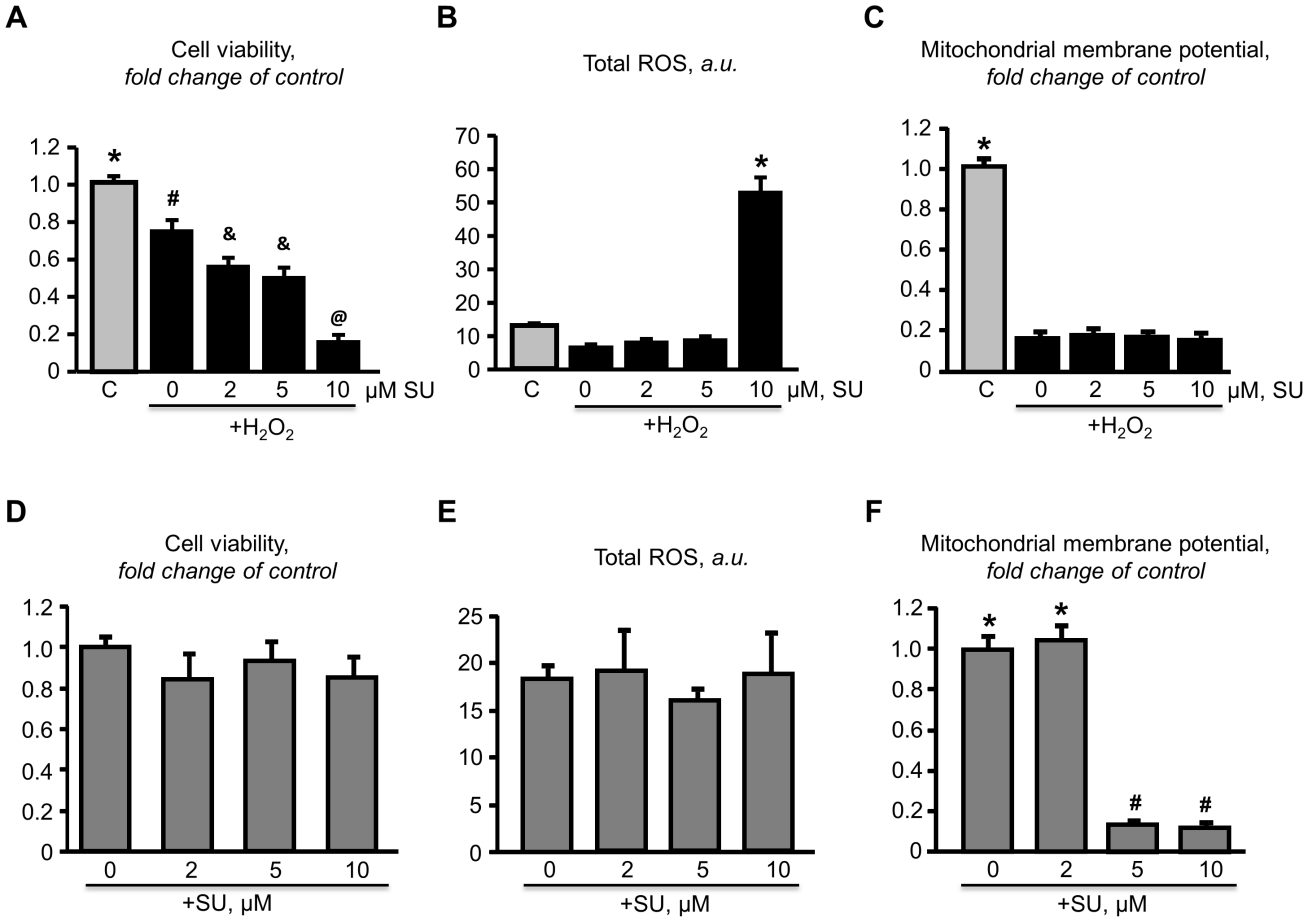
Effect of SU3327 on H9C2 cells exposed to H_2_O_2_. Cardiomyocytes were pretreated with JNK inhibitor SU3327 (SU), then treated with 75 µM H_2_O_2_ (***A,B,C***). Cardiomyocytes were treated with SU only (***D,E,F***). Cell viability (***A,B***), total ROS (***B,E***), mitochondrial membrane potential (***C,F***). *, #, &, @: significantly different from the other indicators (*P*<0.05). n = 6 per group.

As shown in (Fig. 7), inhibition of JNK in H_2_O_2_-treated H9c2 cardiomyocytes increased the activation of both p38 and ERK in a dose-dependent manner. SU3327 exerted detrimental effects on cell viability and increased total ROS in cardiomyocytes subjected to H_2_O_2_-induced stress. Notably, the p38 inhibitor BIRB796 alleviated the detrimental effects of SU3327. However, inhibition of p38 did not promote recovery of the decreased the mitochondrial membrane potential caused by SU3327. In addition, SU3327 did no effect on cell viability, the mitochondrial membrane potential or total ROS in cells that were not subjected to oxidative stress (Fig. 8).

## Discussion

This study aimed to clarify the controversy regarding the effects of JNK inhibition on cardiac IR. For the first time, we demonstrated that the specific inhibition of JNK by SU3327: i) aggravated the recovery of heart function and respiration of mitochondria during IR, ii) inhibited JNK and activated cytosolic and mitochondrial p38, and iii) stimulated interaction of JNK and p38 with complex III of ETC.

JNK, a stress-related MAPK, is involved in apoptotic cell death, in addition to inflammatory responses and cell proliferation, and thus has therapeutic potential in the treatment of inflammatory diseases and cancer. Since the most widely used pharmacological JNK inhibitor SP600125 became available [29], many studies have provided contradictory data on the inhibition of JNK. These conflicting observations could result from the different roles of JNK, which targets both pro- and anti-apoptotic pathways [30]. Moreover, many inhibitors of cellular kinases, including JNK inhibitors, were found to be rather non-specific due to their targeting of the well conserved ATP-binding site [31] making the conclusions derived from their use questionable. Indeed, SP600125, one of these ATP-competitive JNK inhibitors, was found to inhibit 13 of the 30 tested kinases with similar or greater potency than JNK isoforms [32]. For instance, AMPK, which plays a crucial role in cardioprotection, was inhibited 56% by SP600125 at 1 µM. According to the updated report, twenty-two kinases out of 69 tested were inhibited by more than 56% [33]. Therefore, it was recommended that use of SP600125 in the investigation of JNK be discontinued. In our study, we used the JNK inhibitor SU3327 [34], which targets the docking site of JNK with JNK-interacting protein-1 (JIP1), rather than the well-conserved ATP-binding site, and, thereby, demonstrates high specificity for JNK. The use of this specific inhibitor would help reduce off-target effects of JNK inhibitors.

Previous studies showed that inhibition of JNK can confer both adverse [35]–[37] and beneficial [11], [38], [39] effects. Inhibition of JNK protected the myocardium from *in vivo* IR [17] and reduced IR-induced cardiomyocyte apoptosis and infarct size [12], [13]. Interestingly, both genetic inhibition and activation of JNK protected the heart against IR-induced cell death [17]. These studies indicate the complexity of JNK signaling; both sustained inhibition and activation elicits cellular protection, probably through different mechanisms. Our study showed that SU3327 actually aggravated post-ischemic recovery of cardiac function, although it reduced LDH activity at the beginning of reperfusion. In addition, the detrimental effects of JNK inhibition on non-ischemic hearts may be due to reciprocal activation of p38; indeed, although SU3327 did not affect JNK phosphorylation, it doubled activation of p38. These results indicate that the role of JNK could be context-dependent [40] and that cross-talk may exist between the JNK and p38 pathways. Our *in vitro* studies of cultured cardiomyocytes showed that p38 inhibition could alleviate the aggravation caused by JNK inhibition, implying that p38 activation may be the main cause of the aggravation. It is notable that recovery of the mitochondrial membrane potential was not observed along with alleviation, although total ROS was significantly reduced. Activation of p38 was also observed in mitochondria isolated from IR hearts treated with SU3327. Surprisingly, hearts perfused with SU3327 during pre-ischemia only (IRSP group) exhibited activation rather than inhibition of JNK. Because SU3327 only targets JIP1-JNK interaction, pathways other than JIP1 may activate JNK. We also observed the highest activation of p38 in the IRSP group. It is tempting to speculate that inhibition of JNK by SU3327 prior to ischemia stimulates a complementary response of both stress-activated protein kinases which remain activated throughout the entire IR period.

In addition, our studies confirm an essential role of JNK in physiological cell signaling; JNK inhibition aggravated the function of isolated hearts treated with IR. JNK isoforms could respond differently to oxidative stress through stimulation survival or death pathways due to the distinct role they play in cell signaling. JNK has 10 isomers expressed from three different genes, and at least three JIPs have been reported. However, pharmacological inhibition of individual isoforms by inhibitors is remarkably difficult [41].

**Figure 9:**
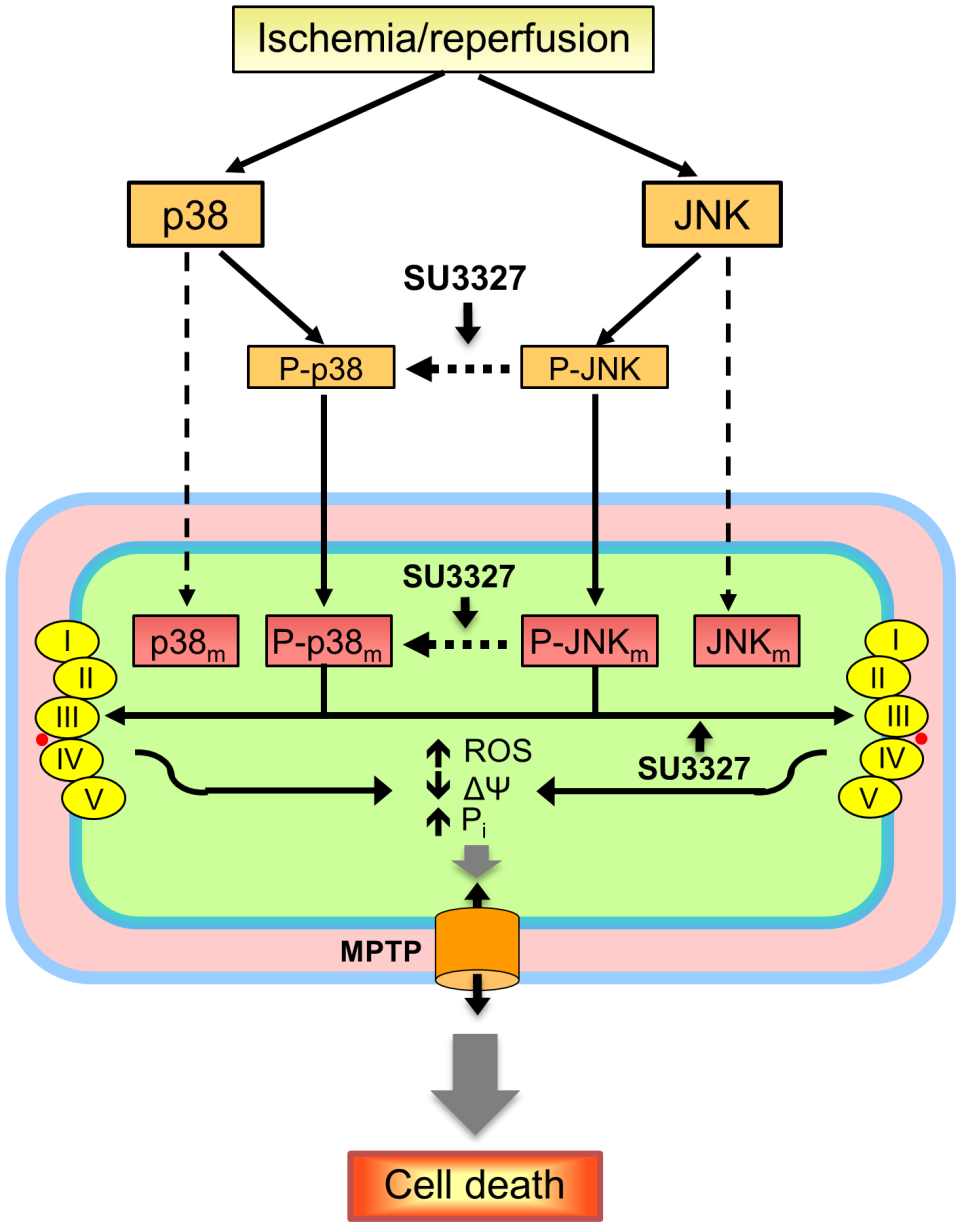
Proposed mechanism of cardiac dysfunction induced by inhibition of JNK. Details of the mechanism are given in *Discussion*. MPTP, mitochondrial permeability transition pore, p38_m_ and P-p38_m_, mitochondrial p38 and P-p38; JNK_m_ and P-JNK_m_, mitochondrial JNK and P-JNK.

The role of mitochondrial JNK in the regulation of mitochondrial function and metabolism is still unclear. JNK is known to interact with Sab, a mitochondrial scaffold of JNK, and the interaction can lead to mitochondrial dysfunction [22]. We found that SU3327 induced inhibition of complexes I and II and opening of MPTPs in IR hearts. CyP-D is known to be a key regulator of MPTP formation, an important mechanism involved in mitochondria-mediated cell death. Although initial studies considered VDAC and ANT to be essential components of the MPTP, subsequent genetic studies questioned this theory. Most recent studies suggested that mitochondrial F_0_F_1_ ATPase, complex V of ETC, may serve as an essential structural component of the pore [42]. We investigated possible interactions of mitochondrial JNK and p38 with CyP-D, VDAC, ANT, and mitochondrial ETC complexes. Although we could detect all of the targets in the mitochondrial fraction, CyP-D, VDAC, ANT as well as complexes I, II and IV were not detected after co-immunoprecipitation (pull-down) with JNK and p38, indicating that these proteins exhibit no physical interaction with the MAPKs. However, complexes III and V were detected after co-immunoprecipitation with JNK and p38. There was no significant difference between treatment groups with regard to complex V, whereas complex III was detected and different among treatment groups, implying strong correlation. This could explain the reduced respiration rate at complexes I and II, but not complex IV. Finally, mitochondria are the major source of ROS produced by complexes I and III in response to oxidative stress [43]–[45]. Inhibition of complex III of ETC due to its interaction with JNK and p38 could initiate ROS production, as evidenced by increased protein carbonylation in the SU3327-treated hearts and cultured cardiomyocytes. High ROS together with depolarization of the mitochondrial inner membrane and ATP depletion can induce MPTP opening thus leading to mitochondria-mediated cell death (Fig. 9). On the other hand, increased ROS can activate p38 through the ASK1/2-MKK4-JNK/p38 pathway [46]. Although JNK can initiate mitochondrial ROS generation [20], the direct relationship between p38 activation and mitochondrial dysfunction is still unclear.

In conclusion, the present study demonstrates that specific inhibition of JNK by SU3327 does not protect the heart from IR injury. Moreover, SU3327 actually aggravates cardiac and mitochondrial dysfunction in hearts exposed to IR, further highlighting the important roles of JNK in cell signaling. SU3327 both stimulates activation of p38 in the cytoplasm and mitochondria and promotes its interaction with the complex III of ETC, suggesting that a reciprocal relationship exists between stress-related MAPKs in response to oxidative stress.
